# Ethical Challenges of the COVID-19 Pandemic: A Japanese Perspective

**DOI:** 10.2196/44820

**Published:** 2023-01-26

**Authors:** Satoshi Kodama

**Affiliations:** 1 Kyoto University Graduate School of Letters Kyoto Japan

**Keywords:** pandemic, Japan, lockdown, disaster preparedness and management, digital technologies, intensive care unit, COVID-19, ICU triage, ethics, emergency preparedness, digital health intervention

## Abstract

This article focuses on how Japan experienced the COVID-19 pandemic. It delineates the various challenges the country faced and the measures the national government took to stop the spread of the infection. The article begins with the author’s personal experience of COVID-19. The second section explains how the Japanese government lacked the legal sanctions to enforce a state of emergency. The third section deals with the current pandemic response as characterized by the increased use of digital technologies to control the spread of the virus. I argue that the lack of effective governance hampered Japan’s timely use of digital technologies. The fourth section will touch on the issues created by the rapid spread of the infection and an increase in the hospitalization rate, focusing on intensive care unit triage and the ethical debates that ensued in Japan. The fifth section discusses the pandemic from the perspective of disaster preparedness and management, exploring the ways the pandemic responses share ethical challenges with responses to other disasters such as earthquakes and typhoons.

## Introduction

This article is a broad overview of Japan’s experience with the COVID-19 pandemic and some of the ethical challenges the pandemic brought about. I want to start with my personal story, interwoven with the development of the COVID-19 pandemic in and outside Japan.

I was on a sabbatical leave in Oxford, United Kingdom, with my family when the news broke about COVID-19, or “the novel coronavirus,” as it was called at the time. “Killer Virus: UK Patients in Isolation” was the headline of the London Metro on January 24, 2020. The day before that, Wuhan in China went into lockdown. About a week later, on 30 January, the World Health Organization declared the coronavirus outbreak a global health emergency. Brexit happened on February 1, 2020. I was still in Oxford. It seemed to me at the time that the United Kingdom was more concerned about Brexit than the news about the new virus.

Then, the news came in about the Diamond Princess cruise ship quarantined in Japan. Not many people outside Japan may remember it now, but the second biggest outbreak after Wuhan happened inside that ship. The cruise ship was staying at the Yokohama Port, not far from the capital city of Tokyo. There were about 3700 people on board, including passengers and crew members. Their quarantine started on February 5 for 14 days, but the number of infected people kept rising, and there was international criticism of the Japanese government’s clumsy handling of the infection. Americans, British, and people of other nationalities returned to their home countries on chartered flights prepared by their governments. When the remaining people finally disembarked on March 1, the number of infections was 712, or about one in five people on board, and the number of deaths was 13 [1].

While the Diamond Princess incident unfolded in Japan, I was in Paris with my family. It was during my daughter’s half-term break from primary school. The timing was perfect because there were few travelers, especially from Asia. But after I came back from France, the situation got more serious. Italy first imposed lockdowns in northern regions in late February, and Italy’s national lockdown followed on March 9. The World Health Organization finally declared COVID-19 a pandemic on March 11 [2].

I was fortunate because my 1-year stay in Oxford ended just as I had planned it a year before. On March 17, my family and I took the Heathrow Express from Paddington Station. The station was almost empty. A week later, on March 23, the United Kingdom started its first national lockdown [3].

Back in Japan, spring was peaceful. Although the schools in Japan were closed from the beginning of March, the government finally declared a state of emergency on April 7 for metropolitan areas such as Tokyo and Osaka and on April 16 for the rest of the country [4].

From here, I will describe and evaluate Japan’s response to the COVID-19 pandemic. The second section explains how the Japanese government lacked the legal sanctions to enforce a state of emergency. The third section deals with the current pandemic response as characterized by the increased use of digital technologies to control the spread of the virus. I argue that the lack of effective governance hampered Japan’s timely use of digital technologies. The fourth section will touch on the issues created by the rapid spread of the infection and an increase in the hospitalization rate, focusing on intensive care unit (ICU) triage and the ethical debates that ensued in Japan. The fifth section discusses the pandemic from the perspective of disaster preparedness and management, exploring the ways the pandemic responses share ethical challenges with responses to other disasters such as earthquakes and typhoons.

## Law Without Sanctions

Japan declared its first state of emergency in April 2020 under “the Act on Special Measures for Pandemic Influenza and New Infectious Diseases Preparedness and Response.” Japan’s National Diet created the Act in 2012 in the wake of the swine flu pandemic (A/H1N1 influenza) in 2009 and revised it in March 2020 to include COVID-19 as a new infectious disease. This revision enabled the then-prime minister Shinzo Abe to declare a state of emergency [5].

Interestingly, Japan’s state of emergency was unlike national lockdowns in Europe and in other Asian countries in that it had almost no penalties for citizens’ noncompliance. The above Act only required citizens to “make efforts to prevent the virus’s spread and cooperate with the government response policies.” Thus, under the state of emergency, people were requested, but not ordered, to stay home unless necessary to go out to buy food and other necessities. The local government also requested restaurants, cafes, and public venues such as public libraries to close temporarily. Furthermore, the government encouraged workers to work from home where possible. People complied; at least enough people stayed home to slow the spread of COVID-19 [6].

The only sanction the Act had for noncompliance was public notification of the name of the noncompliant businesses. In the beginning, many pachinko parlors, gambling houses popular in Japan, did not comply with prefectural governors’ requests to close down. Then, several governors decided to name and shame these pachinko parlors by listing their names on the web. Initially, it worsened the situation because those who wanted to go to a pachinko parlor just checked the local government’s website to locate the still open businesses and went there to have fun. But most parlors complied in the end [7].

The National Diet subsequently revised the Act on Special Measures for Pandemic Influenza and New Infectious Diseases Preparedness and Response in December 2020 to impose fines for such disobedience by businesses. However, most restrictions of liberty lacked penalties. Similarly, COVID-19 vaccination was not mandatory but recommended. Under the current Immunization Act, people only have an “obligation to make efforts” to receive necessary vaccinations. More than 80% of the population have received at least two shots of the COVID-19 vaccine at the time of writing [8].

Comparing the total number of cases and deaths in Japan and Germany, Japan has fared relatively well without resorting to mandatory measures [9]. This is one of the most distinctive features of Japan’s pandemic response, for which an interesting general explanation exists. According to John Haley, an American professor of comparative law, Japan has always been a “society of law without sanctions.” His 1982 article, “Sheathing the Sword of Justice in Japan: An Essay on Law Without Sanctions,” is somewhat outdated, but the main claim is still persuasive. He contends that Japan’s administration often lacks legal sanctions to enforce its orders. Even with sanctions, the administration prefers informal, extralegal means of handling matters because the judiciary is slow and ineffective. Here, I quote one paragraph which characterizes the current pandemic response in Japan [10]:

A legal order without effective formal sanctions need not grind to a halt. Legislators, bureaucrats, and judges may continue to articulate and apply (...) new rules and standards of conduct. The norms thus created and legitimized may have significant impact. To the extent no legal sanctions apply, however, their validity will depend upon consensus and thus (...) become nearly indistinguishable from nonlegal or customary norms. As to those norms the community accepts as necessary or proper, the absence of legal sanctions is likely to produce extralegal substitutes and to reinforce the viability of preexisting means of coercing behavior. Thus the legal order relies increasingly upon community consensus and the viability of the sanctions the community already possesses.

In a word, what Haley says is this: the government in Japan sometimes proposes rules without legal sanctions, but if the public likes them, they attach social or moral sanctions to them, and the regulations become effective.

It is ideal if a society can contain the spread of infection without harsh legal punishment. If all it needs is a name-and-shame tactic to keep people at home and businesses closed, that seems much better than police officers working hard to collect fines from noncompliant people. But reliance on informal sanctions has downsides as well. Social sanctions are open to abuse and difficult to control. In Japan, for example, some vigilantes verbally abused those who did not wear masks in public spaces, damaged facades of restaurants that remained open despite the local governments’ requests to close, or scratched cars which they thought came from outside the prefecture [11]. This abuse of social sanction was one of the main problems Japan faced.

Another problem arising from weak legal sanctions relates to vaccine passports. The domestic use of vaccine passports in Japan has not been successful. Since the official launch of the app on December 20, 2021, only 4.8 million people, or less than 4% of the population, have proof of their vaccine status issued through the official app (as of March 13, 2022). Vaccine passports have not been valuable to people, mainly because the government does not strictly control citizens’ movement with legal sanctions. The vaccine passport has value only if it is legally necessary to show it on entering restaurants or other public places, which was not the case in Japan. The central government allowed businesses and local governments to decide how to use vaccine passports to their advantage. Consequently, many vaccine passport apps were developed, but only a small minority installed them. This case illustrates that the mere introduction of new digital technologies is not enough; there need to be rules set up to mandate the effective use of the technology.

## Use or Nonuse of Digital Technologies

Many still regard Japan as one of the leading countries to develop new digital devices. Sony and Nintendo are good examples. However, the COVID-19 pandemic revealed that the public health infrastructure urgently needs updating, and that the government needs to improve at developing new digital devices.

Local public health centers did much work to contain the spread of COVID-19 in Japan, at least initially. There are around 500 public health centers in Japan. They did most of the contact-tracing work. They also acted as the gatekeepers so that the hospitals could focus on treating moderate and severe COVID-19 cases without being overwhelmed with patients. However, it soon turned out that they worked using outdated infrastructure.

The daily use of fax machines hampered the effective operation of public health centers with the surge of infected people. Fax machines were popular in the 1980s and 1990s but gradually disappeared with the rise of the internet. However, they are still used by health care institutions and the police [12]. Even after the introduction of the Health Center Real-time Information-sharing System on COVID-19, a new surveillance system created for COVID-19, many medical doctors still sent documents by fax, and the staff at the public health centers typed in the information to the System. This case again illustrates that a mere introduction of new digital technologies is not enough. Without enforcement rules, the effective use of the technology is hampered.

Similar lessons arose with the Covid-19 Contact-Confirming Application (COCOA) developed and used in Japan since June 2020. Initially, there were glitches in the app, which hindered its smooth introduction to the public. However, what was more problematic was its voluntariness, in two senses. First, people were not required to install the app. Second, people infected with COVID-19 did not need to put their information in the app. Such an app only works well if those infected and those who had close contact with them have installed the app, and the infected person puts the information into it. As of March 2022, almost 34 million people, or 27% of the population, downloaded the app, and around 620,000 people, or 11% of the total number of infections, registered their status in the app [13]. The government decided to stop using the app by the end of 2022. Once again, good digital technology needs good governance to work well.

In September 2021, the national government set up the Digital Agency to facilitate the digital transformation of the local and national government’s administration [14]. It remains to be seen how it will incorporate the lessons learned from the COVID-19 experience.

## ICU Triage and Other Priority Settings

In March 2020, the so-called Bioethics Study Group in Japan proposed a protocol for distributing artificial respirators during the COVID-19 patient surge [15]. This proposal allowed the redistribution of respirators, meaning moving respirators from dying patients to more promising patients. In such cases, according to the protocol, it is desirable, but not mandatory, that the patient or their family agree to have the respirator withdrawn.

The proposal called for establishing triage guidelines by public bodies, but the call did not materialize. This was due to both complacency and reluctance. Some thought Japan had an excellent health care system, and there was no need to prepare such triage guidelines. Others were reluctant to create such policies, fearing the public would severely criticize them.

A severe shortage of ICU beds and health care staff happened in Osaka and elsewhere when the so-called fourth wave of COVID-19 infection hit Japan in the spring of 2021. Hospitals had to manage primarily for themselves, and what kind of rationing took place in hospitals has yet to be researched.

Both government and business in Japan seem good at improving efficiency (*kaizen*) but need to improve in making hard choices involving questions of life and death and fairness.

In May 2021, upset by the languid pace of vaccination before the Summer Olympic Games, then-prime minister Yoshihide Suga promised 1 million vaccine shots daily. The government soon achieved the target in June, partly because the government set up extra venues for immunization. But in the process, fairness was compromised. Initially, people aged 65 years and older and those with underlying conditions were the second highest priority group for vaccination, after health care professionals. However, before most of them received their first shot, those under 65 and even university students started receiving the vaccination. Naturally, some people were angry at this prioritization of efficiency over fairness.

Another example is the government’s distribution of face masks. In April 2020, when there was a scarcity of face masks in the market, then-prime minister Abe decided to distribute masks to the general population. Apparently, “Two masks for every household” was his idea of fairness. His decision respected equality in a way but neglected the size of the household or the actual needs of people [16].

Equality and liberty are two essential values. If we need not compromise them when we try to improve efficiency, it is all for the good. However, when push comes to shove, scarcity may demand us to make hard choices. In addition, if we must make such decisions, we had better make them fairly. Japan has been making these decisions without respect to fairness, prioritizing efficiency but in fact sometimes acting unfairly as a result.

## Need for Pandemic Ethics

A general lack of emergency planning in Japan is the topic of this final section. One typical answer to the famous trolley problem (would you kill 1 person to save 5 lives by diverting the runaway trolley, or let it run its course and let the 5 die?) is, “that wouldn’t happen in real life” or “we should prepare in advance so that such incident wouldn’t happen.” This type of answer allows us to avoid making a hard choice but does not tell us what to do if such an emergency happens.

Inoue [17], a legal philosopher, argued that the Japanese government’s handling of the COVID-19 pandemic confused risk management and crisis management. In risk management, one identifies possible risks, assesses them, and finds ways to minimize them. By contrast, in crisis management, one prepares for an emergency to minimize harm when a crisis happens despite such risk management. Thus, making sure that the trolley situation will not happen is a task for risk management, while devising ways to minimize harm in the eventuality of the trolley situation is a mission for crisis management. Inoue’s point was that the government avoided discussing crisis management because it was complacent and satisfied with its high standard of risk management [17]. As with the trolley problem, they thought a crisis would not happen.

This way of thinking is odd, given that Japan is a disaster-prone country. Typhoons and earthquakes are everyday experiences for Japanese people, and they learned the hard way from the 2011 Tohoku Earthquake and Tsunami that risk management was not good enough. Since then, the government and the public have discussed disaster preparedness and crisis management more. However, there appears to be a conceptual barrier to seeing the pandemic as a disaster. Japan’s law treats infectious diseases differently from natural disasters. Moreover, both ordinary people and the media continue to see the restrictions of individual liberty under the public health emergency as akin to wartime restrictions of freedom, which is anathema to most Japanese people [18].

However, as we are likely to experience both another pandemic and other large-scale disasters in the coming decades, we need to discuss pandemic ethics, or more generally, disaster ethics, where the need for coercive measures and fair allocation of resources comes to the fore. Without going into detail, I want to raise one issue related to pandemic ethics, that is, whether we should have two ethics—one for normality and another for crises. Zack [19], an American philosopher, argued that we should have only one ethics. Thus, if saving all lives is the right thing to do in ordinary times, we should adopt that policy when making emergency plans.

This way of thinking makes a life-and-death triage nearly impossible in a crisis. But it is somewhat understandable if we think about the following problem. Suppose we have different ethics, or more precisely, a different set of action-guiding principles in an emergency. In that case, such ethics may seep in and alter the ethics in ordinary times. For example, suppose an ICU triage is justified in a pandemic. Such triage might justify giving a lower priority to older people because of their poor survival rate. Subsequently, people might start to think that a similar resource allocation is also permissible in a nonpandemic situation. The question is, can we practically keep the two ethics separate? The Japanese people’s reluctance against publicly discussing crisis management may partly stem from such worries. This aspect of pandemic ethics merits more discussion.

## Conclusions

I have given a broad overview of Japan’s experience with the COVID-19 pandemic and some of the ethical challenges the pandemic brought about. In summary, two kinds of reluctance, the reluctance to impose legal sanctions and the unwillingness to discuss ethics in a crisis, stood out in Japan’s response to the pandemic. In conclusion, I would like to return to my personal experience.

I have been lucky during the pandemic because none of my family have been personally seriously affected by COVID-19. The pandemic has not severely disrupted my daughter’s primary school education except for the occasional online classes that lasted no longer than 2 months in total. My office at the university campus was accessible most of the time, so I usually gave my online classes from my office and did research there. I have had only 2 polymerase chain reaction tests in 2 years, with negative results. I had 2 Moderna vaccine shots in the summer of 2021 and another one on March 2022. A handful of my students reported having caught COVID-19 or being in close contact with someone with COVID-19. However, according to my COCOA app, I have never been in close contact with anyone with COVID-19 for 574 days since the pandemic began. After returning from the United Kingdom, I have not travelled abroad and have only been to Tokyo a few times (as of March 14, 2022). Apart from that, my academic life was not severely disrupted.

University students were not so lucky. At Kyoto University, where I work, most courses were given online until October 2021. That means they could not attend classes in person for one and a half years and could not socialize with their friends. Some students adjusted to the so-called new normal better than others, but younger students, fresh out of high school, suffered the most because they did not have friends at the university and did not know how to cope with the new environment.

Could Japan have done better? It could have, but only if Japan had used digital apps such as COCOA and vaccine passports much more effectively. To make that happen, Japan needed not only effective digital technologies but also effective governance, which may likely involve legal enforcement. There is also a need for empirical research that addresses the general public’s concerns about fairness in resource allocation. An international comparison of COVID-19 responses in Japan and other countries will enable us to be better prepared for the next pandemic than we were for the present one [20].

## Abbreviations

COCOA: Covid-19 Contact-Confirming Application
ICU: intensive care unit
